# Low baseline serum albumin as a predictor of anemia in chronic hemodialysis patients

**Published:** 2015

**Authors:** Behzad Heidari, Hassan Taheri, Karimollah Hajian-Tilaki, Mehdi Yolmeh, Roghayeh Akbari

**Affiliations:** 1 Department of Internal Medicine, Ayatollah Rouhani Hospital, Babol University of Medical Sciences, Babol, Iran; 2 Department of Social Medicine, Babol University of Medical Sciences, Babol, Iran; 3 Department of Internal Medicine, Division of Nephrology, Shahid Behesti Hospital, Babol University of Medical Sciences,Babol, Iran

**Keywords:** Hemodialysis, Anemia, Serum Albumin

## Abstract

**Background:**

Inflammatory process has a substantial contribution in the development of anemia in chronic hemodialysis patients. Low serum albumin in hemodialysis patients is considered a marker of inflammation. The present longitudinal study aimed to determine the relationship between low baseline serum albumin and future development of anemia.

**Methods:**

The population of this study consisted of all patients on standard maintenance hemodialysis for at least three months or longer. Patients were classified as high or low serum albumin level (≥ or < 3.9 gr/dl). All patients received the standard treatment of anemia. The main objective of this study was to compare the prevalence of anemia defined as hemoglobin levels < 11 gr/dl between the two study groups at the end of the study period.

**Results:**

A total of 82 patients (50% females) with mean±SD age of 55±16.8 years and mean dialysis duration of 5.2±4 years were followed-up for an average period of 10±1 (range, 8-11) months, however 48 patients with high serum albumin and 24 patients with low serum albumin group completed the study. At baseline, the two groups were similar regarding hemoglobin (9.8±1.2 vs 9.16±1.6 gr/dl, P=0.95) levels. At endpoint, prevalence of anemia in high albumin group was significantly lower than the low albumin group (50% versus 83.3% P=0.005).

**Conclusion:**

The findings of this study indicate that low serum albumin in hemodialysis patients is a predictor of anemia indicating unresponsiveness to conventional treatment of anemia.

Inflammatory process has a substantial contribution in the development of anemia in chronic hemodialysis patients through unresponsiveness to erythropoietin stimulating agents (ESA). (1, 2). Several markers including serum C-reactive protein (CRP), erythrocyte sedimentation rate (ESA) and serum albumin are considered as measures of inflammation, and are usually used for the assessment of inflammatory state (3-5) and prediction of treatment response (3, 6). Among the parameters of inflammation, low serum albumin is of particular importance, because it is a reflection of nutritional status and inflammation (7, 8) as well as a marker of protein-energy wasting and a component of malnutrition-inflammation complex (MIC) (8, 9). Low serum albumin in hemodialysis patients is also a predictor of cardiovascular disease (CVD) and mortality (8, 9). Low serum albumin is associated with lower survival rate (9, 10). Anemia in hemodialysis patients is an independent risk factor for survival, quality of life and increased morbidity (11, 12).

The relationship between anemia and inflammatory process in hemodialysis patients as assessed by high serum CRP has been addressed in several studies (13-16) but the data regarding serum albumin and anemia in hemodialysis patients are scarce. We performed the present longitudinal study to determine the relationship between baseline serum albumin and future anemia in hemodialysis patients.

## Methods

The population of this longitudinal study consisted of all patients who were on standard maintenance hemodialysis for at least three months or longer in the hemodialysis center of Shahid Beheshti Hospital of Babol University of Medical Sciences. Patients with coexistent inflammatory diseases, infectious diseases, temporary hemodialysis with catheters and patients who received blood transfusion during three months prior to the study were excluded. The proposal of this study was approved by the Ethics Committee of Babol University of Medical Sciences, Babol, Iran. All study patients were followed-up from September 2013 to October 2014. Data were collected by follow-up regular clinical examination and laboratory tests, interview, and review of the medical records. Serum albumin, total protein, ferritin, transferrin, iron, hemoglobin, hematocrit, platelet count, calcium, phosphate, alkaline phosphatase, parathyroid hormone, fasting blood sugar, cholesterol, triglycerides, creatinine, blood urea nitrogen BUN, were measured regularly over the study period. All laboratory tests were performed in a single laboratory. Anemia was confirmed by hemoglobin levels < 11 gr/dl. Patients were classified as high or low serum albumin group according to serum albumin level of ≥ or < 3.9 gr/dl. All patients received the standard treatment of anemia in hemodialysis patients by oral or intravenous iron as well as ESA as appropriate. The main objective of this study was to compare the prevalence of anemia between the two study groups at the end of the study period. Chi- square test was applied for comparison and SPSS software Version 18 was used for analysis.

## Results

A total of 82 patients (50% females ) with mean (± SD) age of 55±16.8 years and mean dialysis duration of 5.2±4 years were followed-up for an average period of 10±1 (range, 8-11) months but 48 patients with high serum albumin and 24 patients with low serum albumin group completed the study.

The causes of the end-stage renal disease (ESRD) were hypertension in 29 (35.3%), diabetes in 22 (2 6.8%) obstructive uropathy in 12 (14.6%), pyelonephritis in 4 (4.8%) and undetermined causes in 15 (18.2%). The mean age of the patients in the two groups was 52±17 years and 58±17 years (P=0.23) respectively. At baseline, the two groups were similar regarding hemoglobin and iron parameters as well as other biochemical variables (table 1). 

**Table 1:** Comparison of hemoglobin and other blood parameters as well as biochemical parameters and the prevalence of anemia in hemodialysis patients over the study period according to low or high serum albumin concentrations

**Variables**	**Baseline values**			**Endpoint values**		
	**Low albumin** **N=24**	**High albumin** **N=48**	**P-values ** #	**Low albumin** **N=24**	**High albumin** **N=48**	**P values** #
Age, year	58±17	52.7±17.2	0.23	57±15	54±16	0.29
Hemoglobin gr/dl	9.1±1.2	9.1±1.6	0.23	9.75±0.93	9.7±1.9	0.20
Serum Iron µg/dl	58.8±52.7	55.4±34.7	0.79	66.1±72.3	61.5±41.7	0.8
TIBC µg/dl	329±37.6	314.6±51	0.18	316±44.9	279±97.9	0.09
Serum Ferritin ng/ml	79.9±81.9	134.8±157.6	0.12	54.7±48.1	1.3.5±111.6	0.10
Serum cholesterol mg/dl	145±34.5	153±42.6	0.84	148±37.2	159±40.8	0.25
Transferrin saturation (%)	20.2±23.5	18.7±22.2	0.84	31.3±31	41.1±17.3	0.37
Serum albumin, mean gr/dl	3.66±0.16	4.06±0.18	0.001	3.6±0.75	4.2±0.25	0.001
Dialysis duration, years	4.4±3.2	5.5±4.5	0.3	5.4±3	6.4±4.1	0.34
Anemia, no (%)	18 (75)	35 (72.9)	0.54	20 (8.3.3)	24 (50)	0.005

Low and high serum album in (< or ≥ 3.9 gr/dl)

# Comparison was performed using student t-test

Eighteen out of 24 patients (75) in the low albumin group and 35 out of 48 (72.9) patients in high albumin group had anemia (P=0.54). At the end of the study period, hemoglobin decreased in low serum albumin by 0.17±1.4 gr/dl (95% confidence interval, -1–0.73 gr/dl) but in the high serum albumin group increased by 0.73 gr/dl (95% confidence interval, -0.24- 1.7) (P=0.15). None of the iron or biochemical parameters in both comparison groups changed significantly as compared with baseline values (table 1). In high serum albumin group however, the prevalence of anemia decreased significantly in the high serum albumin group (50% versus 83.3%, P=0.005).

## Discussion

The findings of this longitudinal study showed that hemodialysis patients with lower concentration of baseline serum albumin had significantly higher prevalence of anemia at the end of the study period indicating lower rate of response to conventional therapy of anemia. Since the two groups were similar with regard to demographic characteristics and the associated factors of iron parameters at both baseline and endpoint stage except mean serum albumin, therefore, improvement of anemia across the comparison group should be attributed to increased concentration of serum albumin which has occurred in high serum albumin group. 

These observations indicate that in hemodialysis patient increase in serum albumin is expected to be associated with greater improvement of anemia and the patients, with low serum albumin are at greater risk of anemia which is in agreement with the results of earlier studies indicating that in hemodialysis patients, low serum albumin is used as a marker of inflammation (7-9). The relationship between anemia and inflammatory process in hemodialysis patients were shown in several studies (11-13). In these studies, patients with high concentrations of serum CRP or low levels of serum albumin had lower hemoglobin levels (2, 13, 16). The results of the present study found a relationship between low serum albumin and anemia, possibly due to hyporesponsiveness to ESA in hypoalbuminemic patients (1, 7, 17). The mechanism by which inflammation affect erythropoiesis has been explained by the increased levels of cytokines particularly interleukin -1, this cytokine increases production of CRP and reduces serum albumin and transferrin synthesis. Low level of transferrin prevents iron transport to the hematopoietic sites and leads to low hemoglobin synthesis as well as hyporesponsiveness to ESA (1, 7, 18, 19). In this study, transferrin did not change significantly concurrent with serum albumin. This may be attributed to small sample size and short duration of the study. Nonetheless, in this study, the presence of hypoalbuminemia in hemodialysis patients predicted future development of anemia, this issue justifies further consideration in the treatment of anemia in hemodialysis patients who have hypoalbuminemia. In particular, both hypoalbuminemia and anemia are associated with higher morbidity and mortality in hemodialysis patients .Thus presence of low serum albumin in these patients not only reduces ESA response but imposes the at greater risk of morbidity and mortality (8-12).

This study has limitations with reference to the measurement of other marker of inflammation like serum CRP and details data about treatment of anemia particularly ESA disages. In addition, we did not collect data about other associated factors of anemia. Therefore, the extent of contribution of low serum albumin alone in the development of anemia or hyporesposiveness to ESA requires further prospective study. However, the longitudinal design of this study can be considered as a strength.

In conclusion, the results of this study indicate that hemodialysis patients with low serum albumin are at greater risk of anemia. And the level of serum albumin can be considered as a predictor of treatment response in hemodialysis patients.

## Notes

**Funding: **This study was supported by a grant (No 9237132) from Babol University of Medical Sciences, Iran and it is a thesis project of Mehdi Yolmeh (No: 419)

## References

[1] Rattanasompattikul M, Molnar MZ, Zaritsky JJ (2013). Association of malnutrition-inflammation complex and responsiveness to erythropoiesis stimulating agents in long-term hemodialysis patients. Nephrol Dial Transplant.

[2] Panichi V, Rosati A, Bigazzi R (2011). Anaemia and resistance to erythropoiesis-stimulating agents as prognostic factors in haemodialysis patients: results from the RISCAVID study. Nephrol Dial Transplant.

[3] Heidari B (2013). C-reactive protein and other markers of inﬂammation in hemodialysis patients. Caspian J Intern Med.

[4] Heidari B (2012). The importance of C-reactive protein and other inﬂammatory markers in patients with chronic obstructive pulmonary disease. Caspian J Intern Med.

[5] Firouzjahi A, Monadi M, Karimpoor F (2013). Serum C-reactive protein level and distribution in chronic obstructive pulmonary disease versus healthy controls: a case-control study from Iran. Inflammation.

[6] Heidari B, Heidari P, Tayebi ME (2007). The value of changes in CRP and ESR for predicting treatment response in rheumatoid arthritis. APLAR J Rheumatol.

[7] Agarwal R, Davis JL, Smith L (2008). Serum albumin is strongly associated with erythropoietin sensitivity in hemodialysis patients. Clin J Am Soc Nephrol.

[8] Kaysen GA (2006). Association between inflammation and malnutrition as risk factors of cardiovascular disease. Blood Purif.

[9] de Mutsert R, Grootendorst DC, Indemans F (2009). Association between serum albumin and mortality in dialysis patients is partly explained by inflammation, and not by malnutrition. J Ren Nutr.

[10] Chung SH, Lindholm B, Lee HB (2003). Is malnutrition an independent predictor of mortality in peritoneal dialysis patients?. Nephrol Dial Transplant.

[11] Casses A, Coll E, Collado S (2009). Anemia in chronic kidney disease and its cardiovascular implications. Med Clin.

[12] Selim G, Stojceva-Taneva O, Sikole A (2007). Association between haemoglobin level and all-cause mortality in haemodialysis patients: the link with inflammation and malnutrition. Prilozi.

[13] Owen WF, Lowrie EG (1998). C-reactive protein as an outcome predictor for maintenance hemodialysis patients. Kidney Int.

[14] Teruel JL, Marcen R, Ocaña J (2005). Clinical significance of C-reactive protein in patients on hemodialysis: a longitudinal study. Nephron Clin Pract..

[15] Wang AY, Woo J, Lam CW (2003). Is a single time point C-reactive protein predictive of outcome in peritoneal dialysis patients?. J Am Soc Nephrol.

[16] Heidari B, Fazli MR, Misaeid MA ( 2014 Nov 8). A linear relationship between serum high-sensitive C-reactive protein and hemoglobin in hemodialysis patients. Clin Exp Nephrol.

[17] Ogawa T, Shimizu H, Kyono A (2014). Relationship between responsiveness to erythropoiesis-stimulating agent and long-term outcomes in chronic hemodialysis patients: a single-center cohort study. Int Urol Nephrol.

[18] Madore F, Lowrie EG, Brugnara C (1997). Anemia in hemodialysis patients: variables affecting this outcome predictor. J Am Soc Nephrol.

[19] Barany P (2001). Inflammation and, serum C-reactive protein and erythropoietin resistance. Nephrol Dial Transpl.

